# Characterisation of high molecular weight hop proanthocyanidins using Analytical Ultracentrifugation

**DOI:** 10.1038/s41598-019-49171-4

**Published:** 2019-09-02

**Authors:** Arthur Gadon, Robert Linforth, Stephen E. Harding, David Cook

**Affiliations:** 1 International Centre for Brewing Science, School of Biosciences, University of Nottingham, Sutton Bonington Campus, Sutton Bonington, Leicestershire UK; 2 Division of Food Sciences, School of Biosciences, University of Nottingham, Sutton Bonington Campus, Sutton Bonington, Leicestershire UK; 3 National Centre for Molecular Hydrodynamics, School of Biosciences, University of Nottingham, Sutton Bonington Campus, Sutton Bonington, Leicestershire UK; 4 Kulturhistoriskmuseum, Universitetet i Oslo, Postboks 6762, St. Olavs plass, 0130 Oslo, Norway

**Keywords:** Biophysics, Biotechnology

## Abstract

We report the novel application of Analytical Ultracentrifugation (AUCF) to characterise the polymeric proanthocyanidin fraction of hops. Extraction of hop samples with 70% acetone (aq) followed by a C-18 Solid Phase Extraction yielded polyphenolic fractions for AUCF analysis. Sedimentation velocity experiments demonstrated the presence of discrete molecular weight bands of proanthocyanidins, as opposed to a continuous distribution of molecular weights. There were 4 such bands for Saaz hop (0.15, 1.1, 2.7 and 4.4S) and 3 bands for Magnum (0.15, 1.6 and 3.0S). The method resulted in a reproducible size (sedimentation coefficient) distribution for replicate runs of the same extract and for extracts prepared from different samples of the same hop variety. Sedimentation equilibrium experiments were then used to fit molecular weight distributions using the new SEDFIT-MSTAR method for the same samples. Thus we report for the first time polymeric proanthocyanidins in hops with molecular weights of up to 100 kDa in Saaz hop (or up to 56 kDa in Magnum). This represents the first application of AUCF to characterise complex fractions of polyphenolics extracted from botanical sources and the methodology developed should find wider application in the study of this diverse and bioactive class of compounds.

## Introduction

Polyphenolic molecules are broadly distributed across the plant kingdom and thus represent an important part of the human diet. Recent studies have demonstrated their beneficial impact on some health conditions, such as urinary tract infections and cystitis^1^. Amongst the polyphenols, proanthocyanidins, (also known as condensed tannins), are often associated with these benefits^2^.

Proanthocyanidins belong to the flavonoid group, and are polymeric chains of flavanols, mostly composed of catechin or epi-catechin subunits. They are present in a wide range of fruits, vegetables and berries, and their molecular size range varies from dimers to larger polymeric molecules upwards of 15 kDa. For example, the average degree of polymerisation (DP) of proanthocyanidins in chokeberries has been reported to be 50 monomeric units^3^.

Polyphenolic fractions extracted from hops have been shown to flocculate yeast and proteins in green beer, and can thus be used to aid beer clarification^4^. This fining activity is linked to the proanthocyanadin content of extracts, as indicated by the acid butanol assay for condensed tannins^5^. In an attempt to characterise the molecular size of the proanthocyanidins fractions extracted from hops, Linforth *et al*.^4^ used a series of molecular weight cut-off spin filters to fractionate extracts. This indicated the presence of molecules greater in size than 30 kDa (equivalent to a DP of >100). However, spin filter fractionation is only a crude method for size characterisation and clearly lacks resolution. Tanaka *et al*.,^6^ likewise reported large amounts of highly oligomeric (>20-mer) proanthocyanidins in their hop bract extract. The oligomers were separated using High-Speed Counter-Current Chromatography (HSCCC) fractionation following ultrafiltration (cut-off >3 kDa). Structural studies using ^1^H and ^13^C NMR indicated that the proanthocyanidins were of the B-type, with flavan-3-ol units that are linked through C-4 to C-8 (or C-6) interflavan bonds.

The analysis of proanthocyanidins presents characterisation challenges, particularly when it comes to the larger polymers^7^. Small polymers, up to the tetramer (DP = 4), are easily fractionated by chromatography^6^ and analysed by MALDI-TOF^8^. However, the analysis of large proanthocyanidins (DP >20) by MALDI-TOF is challenging. Monagas *et al*.^8^ commented that MALDI-TOF-MS is not well-suited to the analysis of highly polydisperse proanthocyanidins, due to the generation of multiply charged ions which makes spectral interpretation difficult. Furthermore they concluded that high molecular weight polymers were challenging with regard to suppression of ionisation, reduction of desorption and in-source fragmentation to form non-covalent ion clusters. More recent analytical developments have focused on characterising the linkage structure of proanthocyanidins using UHPLC-MS based methodologies^9,10^. Furthermore, Longo and co-workers have demonstrated the presence of novel cyclic B-type hexameric proanthocyanidins in red wine using Isotopic Exchange HPLC-HRMS/MS^11^.

Chromatographic methods are limited in terms of accurate analysis of molecular weight due to the absence of appropriate calibration standards^12^. Hence there is a need for improved methodology for the analysis of large proanthocyanidin polymers, particularly those species with DP >4. Improved analytical methodologies will help to increase our understanding of the biological roles of these complex compounds^13^.

Here we present the development of a novel analytical technique for the characterisation of high molecular weight proanthocyanidins using Analytical Ultracentrifugation (AUCF) to characterise the molecular composition of polyphenolic hop extracts. AUCF is a hydrodynamic method which enables the real-time monitoring of macromolecules in solution^14,15^. The principle of this technique is to use a high centrifuge velocity in order to move compounds within a solution through a cell. This transport and the signals acquired are dependent on molecular weights and to a lesser extent molecular conformation. The molecular weight of polymers can thus be estimated from hydrodynamic principles, independently of the need for accurate mass calibration standards. Furthermore, the inherent separation and analysis capability of AUCF – without the need for a separation matrix (column or membrane), and its absolute nature makes it extremely promising for characterising complex systems like polyphenols. This technique is usually applied to the characterisation of purified macromolecules or simple polymer mixtures^16^ and the present study represents the first attempt to apply this technique to analyse complex mixtures of polyphenolics such as are present in fractions extracted from plant materials, taking advantage of recent advances in analysis software such as the SEDFIT-MSTAR procedure of Schuck, Harding and co-workers^17^.

## Materials

### Hop samples

Two hop varieties (Magnum, Saaz) were purchased from the Hop Shop (Plymouth, UK), as whole dried cones. Two further samples of Saaz hop were kindly supplied by Barth Innovations Ltd. (Tonbridge, UK), as pellet and whole dried cones (Saaz 2).

Beers for fining assays were prepared using Young’s Harvest Pilsner Lager home-brew kit, together with dried light malt extract (The Hop Shop, Plymouth, UK), and granulated sugar (Sainsbury’s supermarket, Nottingham, UK). Fermentation was with Nottingham ale yeast (Lallemand).

Acetone, ethyl acetate, methanol, hydrochloric acid, n-butanol, ferric ammonium sulfate were purchased from Sigma Aldrich (Dorset, UK). Milli-Q water (>18 MΩ/cm) was used in the solid phase extraction (SPE) fractionation method.

## Methods

### Extraction

The hops were milled using a Perten 3600 lab mill (Hägersten, Sweden). Hop powder (10 g) was then mixed with 100 mL of acetone/water (variable ratio, v/v), on a Thermo Denley Spiramix roller-board (Loughborough, UK) for 15 min, and subsequently filtered through Whatman No1 filter paper. The acetone was evaporated using a Buchi R-II rotary evaporator (Flawil, Switerland) at 45 °C. The extract was then chilled overnight at 4 °C, before centrifugation at 4000 rpm using a Thermo Jouan CR3i centrifuge (Loughborough, UK) for 10 min and the residue discarded.

### Acid butanol assay for proanthocyanidins content

Acid butanol solution was prepared from 95 mL of n-butanol with the addition of 5 mL of concentrated HCl. 2% ferric ammonium sulfate (FeNH_4_(SO_4_)_2_ ·12 H_2_O) was prepared by adding 1 g of ferric ammonium sulfate into 50 mL of 2 N HCl. For each assay, acid butanol reagent (3 mL) was combined with 25 µL of the hop extract sample and 100 µL of the iron reagent solution in a screw-capped glass tube. Tubes were heated at 90 °C for 30 min. Once cooled, absorbance was measured at 550 nm using a Genesys 10S Vis spectrophotometer (Thermo Scientific, UK). Readings were corrected by subtracting the absorbance of a blank containing only extraction solvent, acid butanol and ferric ammonium sulfate in the same proportions as used in the assay.

### Beer production for assay of fining activity

Green beer for fining was made in 25 L polypropylene fermentation bins using one can of the Young’s Pilsner home brew beer kit. The can contents were dissolved in four litres of boiling water. Granulated sugar (500 g) and dried malt extract (500 g) were added before the mix was made up to 23 L with cold water prior to yeast addition. Beer was used for fining experiments after a minimum of 6 days of fermentation at 20 °C.

### Beer fining assay

Once the beer had been racked and chilled to 4 °C, 500 mL of beer was filled into a 600 mL beaker. Hop extract (4 mL/L) was introduced into each beaker and the contents mixed using a Yellow line IKA OST20 overhead paddle stirrer at 500 rpm for 30 sec. After mixing, the beaker contents were transferred to individual 1 L polycarbonate graduated Imhoff cones (Nalgene: 13 mm closure size, 106 mm diameter). Sediment depth was recorded 2 h and 24 h post-fining. Beer haze (25° and 90° scatter) was measured using a turbidity meter (Haffmans Vos Rota 90/25; Pentair, Germany) at 4 °C 24 h post-fining and after centrifuging samples at 500 rpm for 15 min to remove residual yeast.

### Solid-Phase extraction (SPE) clean-up of extracts for AUCF analysis

Hop extracts, prepared as described above, were fractionated using Sep-Pak C18 Vac 6 cc solid-phase extraction cartridges (Waters, Elstree, UK). The cartridges were equilibrated with methanol and then conditioned with deonised water. Hop extract (5 mL) was then loaded onto the cartridge and washed twice with water (2 × 10 mL) and twice with ethyl acetate (2 × 10 mL). The sample was then eluted with 5 mL of methanol. This procedure was based on the methodology of Sun *et al*.^18^.

### Analytical ultracentrifugation (AUCF), sedimentation velocity analysis

Sedimentation velocity experiments were performed using an Optima XL-I analytical ultracentrifuge (Beckman, Palo Alto, USA) employing Rayleigh interference optics. Methanolic fractions of the hop extract at either 6, 10, 12 or 24-fold dilution (395 µl of sample, and 405 µL methanol reference) were injected into the 12 mm double sector titanium cells with sapphire windows. The 6-fold dilution was run twice in every AUCF experiment. The cells were loaded into the rotor which was spun at 45000 rpm (20.0 °C). Scans were taken at 5 min intervals for an acquisition of 200 scans minimum for each cell.

### Sedimentation equilibrium

Sedimentation equilibrium experiments were performed using the Beckman (Palo Alto, USA) Optima XL-I analytical ultracentrifuge and the Rayleigh interference system. Double-sector titanium cells of 20.0 mm optical path length with sapphire windows were filled with 140 µl of hop extract at 40-fold dilution (sample sector) and 140 µL of reference solvent (methanol) in the reference sector cell. Experiments were performed starting with a low centrifuge speed of 20000 rpm before moving to higher speeds. Once equilibrium was reached at each speed the rotational speed was increased by increments of 5000 rpm, up to a maximum of 40000 rpm for the final scans. Scans were taken every hour, until the equilibrium state at a given speed was reached (the total run time for experiments was between 4–5 days due to the considerable time required to reach equilibrium at each different speed). Samples were analysed at a low concentration (40-fold diluted in methanol) to reduce non-ideality effects^17^.

### AUCF data processing

Sedimentation velocity experimental data were analysed in the SEDFIT algorithm of Schuck^19^ using the c(s) processing^20^ and expressed using the sedimentation coefficient distribution c(s) vs s where s is the sedimentation coefficient in Svedberg units (S = 10^−13^ sec). The sedimentation coefficient distribution is a measure of the size distribution but is influenced by shape. Sedimentation equilibrium data were analysed in SEDFIT-MSTAR^17^, particularly useful for heterogeneous systems. It is based on the M* function of Creeth and Harding^21^ and uses a smart-smooth algorithm for generating distributions of molar mass c(M) vs M. Although less resolving (due to the lower speeds) distributions are absolute (i.e. not requiring assumptions over conformation). The two methods (sedimentation velocity and equilibrium) provide, as we now demonstrate, a powerful combination for the analysis of heterogeneous systems such as the polymeric proanthocyanidin system under analysis here.

## Results

**Figure 1:**
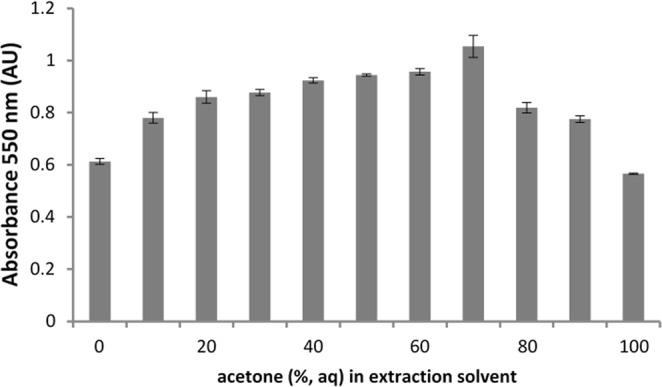
Absorbance values at 550 nm after hydrolysis of Saaz hop extracts using different proportions of acetone in water for the polyphenolic extraction. Data are the mean ± standard deviation of 3 replicate determinations.

Extraction of proanthocyanidins (PA) from hop material using different proportions of acetone in water yielded a series of extracts. These were analysed for their proanthocyanidin content using the acidic butanol assay. Hydrolysis of proanthocyanidins under these conditions produces anthocyanidins which were measured in a spectrophotometer by absorbance at 550 nm. All hop extracts generated the red magenta colour typical of proanthocyanidin cleavage to anthocyanidins. A range of absorbance values were produced, ranging between 0.6–1.02 (Fig. 1). Absorbance values broadly correlate with the amount of proanthocyanidins present but do not yield any information on their molecular weight range^13^.

Extraction of hops with 70% acetone (aq) yielded the highest level of proanthocyanidins. Furthermore, fining performance in green beer (post-fermentation) was likewise found to be optimal at this solvent ratio. Optimal beer fining is determined as the point at which there is minimal haze present in the beer and at which there is a substantial and relatively compact amount of sediment (Table 1). An extraction ratio of 70% acetone was thus selected for the preparation of all further hop proanthocyanidin extracts.

**Table 1:** Flocculation activity and haze results of fined green beer (4 °C) using extracts from different solvent extractions at 4 mL/L.

% Acetone (aq) Extract	Sediment height (mL)		Haze measurement	
	2 h	24 h	H90	H25
0	6.25	6	17.3	27.8
10	6	6	12.7	21.7
20	6.25	6	15.7	25.7
30	6.25	6.5	12.2	20.2
40	6.5	6.5	10.4	18.3
50	6.5	6.25	10.4	18.1
60	6.5	6.5	10.9	19.3
70	6.5	7.25	9.4	17.3
80	6	6	10.9	19.0
90	6	7.25	12.1	21.9
100	6.25	7.5	18.0	28.8

Haze analysis was performed after 24 h of clarification.

H90 = 90° scatter; H25 = 25° scatter;

### Development of an AUCF technique for the analysis of proanthocyanidin extracts from hops

Preliminary AUCF experiments with crude acetone/water extracts did not yield data that could be processed by the AUCF software, presumably due to the wide range of material present within the sample. A C18 Sep-Pak SPE clean-up method was developed (based on that reported by Sun *et al*.^18^ for the separation of grape and wine proanthocyanidins) in order to separate proanthocyanidins from other material. The methanolic fraction eluted from the C18 cartridge was hydrolysed and the acid butanol assay showed a 90% recovery of the PA that was present in the original crude extract (data not shown). The resulting fraction is a clear, non-viscous solution, with a colour ranging from yellow to brown depending on the concentration and the hop variety. The cleaned up fractions proved suitably pure for characterisation by AUCF.

### AUCF data processing parameters: specific volume, solvent density and frictional ratio

The partial specific volume of a substance is represented by the ratio of the substance’s anhydrous volume to its anhydrous mass, and is a key parameter for the processing of the analytical ultracentrifuge data. Tanaka *et al*.^6^ and Linforth *et al*.^4^ each reported that the proanthocyanidins in hops are mainly comprised of catechin and epicatchin subunits. Based on this, the partial specific volume was calculated to be 0.706 mL/g (according to the method of Durchschlag and Zipper^22^). The density of the solvent used for the processing was that of pure methanol at 20.0 °C.

In the SEDFIT procedure^20^ sedimentation coefficient distributions are generated from the evolution of the concentration distribution in the ultracentrifuge cell with time, to give the derived g(s) as a function of s distribution. Although the sedimentation coefficients of discretely resolved components are unaffected, the distribution can be influenced by diffusive broadening. The c(s) as a function of s method in SEDFIT provides a correction for this by evaluating a parameter known as the frictional ratio. The frictional ratio could not be determined from the literature, because the sample studied is not composed of only one molecular species. Instead a (weight average) frictional ratio was thus determined by iterative fitting using the software and applied to all species. All experiments showed slightly different frictional ratio values between samples, but, a consistent value was typically obtained across a dilution series for a given hop extract.

### Sedimentation velocity (SV) AUCF analysis of hop extracts

**Figure 2:**
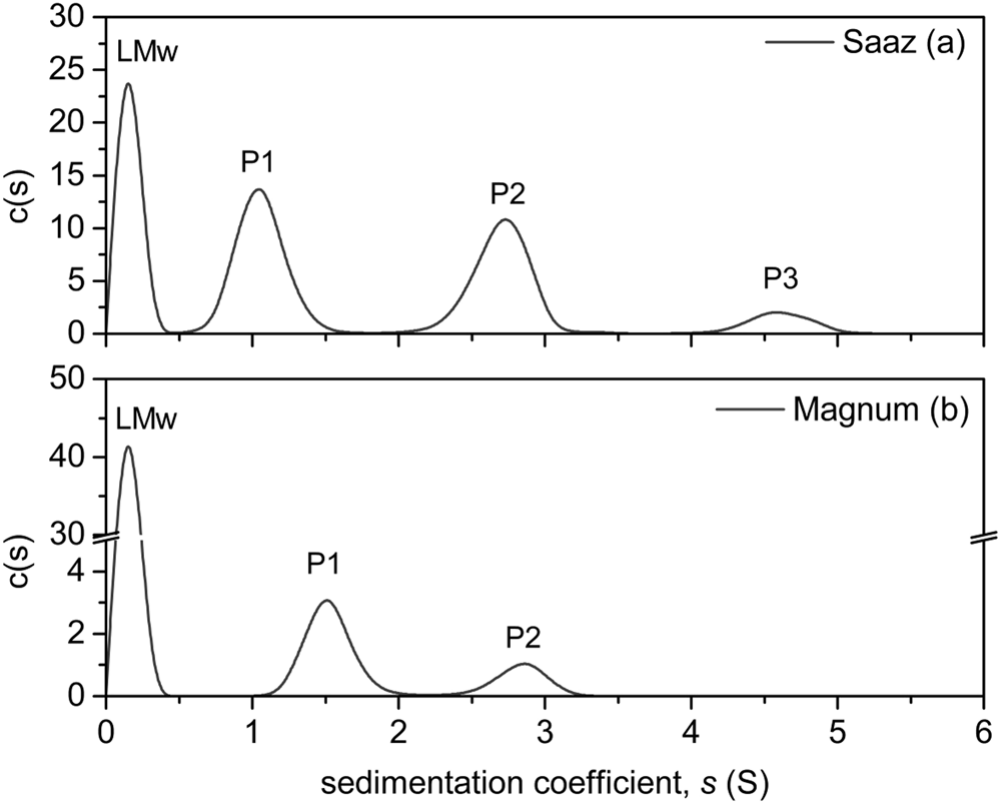
Sedimentation velocity c(s) profiles for Saaz (**a**) and Magnum (**b**) hop extracts (in methanol, 6-fold dilution) after C18 fractionation. Centrifugation velocity 45000 rpm, temperature 20.0 °C. Peaks are labelled LMw (Low Molecular Weight), P1-P3 for cross-reference with corresponding data in Table 2 and Fig. 3.

Sedimentation velocity is an analytical method which measures the rate at which molecules move in response to a high centrifugal force. This force depletes the compounds from the meniscus region (close to the centre of the rotor) ultimately forming a pellet at the outside of the cell. This transport rate is expressed in terms of sedimentation coefficient values, measured in Svedbergs. The sedimentation velocity experiments showed a heterogeneous weight distribution for the polyphenolic extracts of both hop varieties (Fig. 2).

The diffusion-deconvoluted model, c(s) provides evidence of different oligomeric states of the hop proanthocyanidin fractions; the profiles have peaks at discrete size ranges and are not continuous. For Saaz, the profile (Fig. 2a) showed 4 main peaks with sedimentation coefficients of 0.15, 1.1, 2.7 and 4.4. The peak with a coefficient of 0.15 corresponds to unresolvable smaller molecules; monomers and metal ions. Proanthocyanidin-rich extracts of Saaz showed an additional high molecular weight peak (P3, at 4.5S) which was not present in Magnum, as well as a higher signal response for polymerised higher molecular weight material (2.7S). Conversely, the Magnum extract gave a higher signal in the lowest molecular weight band.

These sedimentation coefficient data correspond with a greater fining activity of Saaz compared to Magnum in beer clarification studies (data not shown), supporting the hypothesis that larger proanthocyanidin polymers would result in larger particles of sediment that settle more efficiently.

A key feature of any analytical technique is the reproducibility of the data obtained. Each sample run consisted of a set of cells running simultaneously with different dilutions of an extract. The results from each cell were obtained and averaged to give the c(s) values (Table 2). These showed minimal coefficient of variation (standard deviation relative to the average) for each c(s) peak in the profile. In addition to this there may be day to day and botanical variation between samples. Three independently sourced samples of Saaz were profiled in separate AUCF runs. These showed similar profiles on each occasion, suggesting consistency of this molecular weight range fingerprint for particular hop varieties (Table 2).

**Table 2:** Sedimentation velocity c(s) profiles for Saaz and Magnum hop extracts.

	c(s) sedimentation coefficient values (S)*				%Area^‡^			
	LMw	P1	P2	P3	LMw	P1	P2	P3
Saaz Pellets	0.15 ± 0.003	1.04 ± 0.08	2.64 ± 0.08	4.46 ± 0.13	33.4	32.5	27.7	6.4
Saaz Whole Cones 1	0.15 ± 0.003	1.12 ± 0.07	2.65 ± 0.06	4.22 ± 0.15	34.0	32.4	27.1	6.5
Saaz Whole Cones 2	0.15 ± 0.005	1.09 ± 0.03	2.70 ± 0.06	4.41 ± 0.17	31.8	34.5	27.2	6.5
Magnum Whole Cones	0.15 ± 0.004	1.56 ± 0.05	2.95 ± 0.08	—	84.9	11.5	3.6	0

*Average peak in Svedbergs ± SD of five determinations based on the dilution series of each sample.

^‡^Absolute peak areas were calculated using integration and then expressed as a percentage of total area of each sample to reflect the proportion of material present in each size range.

P1 to P3 are the different molecular size peaks on the c(s) profiles in Fig. 2. Speed 45000 rpm, temperature 20 °C.

The sedimentation velocity technique appears to be highly reproducible, both within and between runs. There also appears to be varietal consistency. This consistency allows us to clearly demonstrate a difference in hop proanthocyanidin molecular weight profiles between different varieties of hops as can be seen in the Saaz and Magnum data (Table 2 and Fig. 2).

In AUCF, sedimentation velocity can define the profile of a mixture and shows the heterogeneous nature of the sample and its global composition. Although related to molecular size, information on molecular weight cannot be directly extracted from sedimentation velocity profiles. However, these experiments do clearly show a discrete set of peaks with distinct differences in molecular size rather than a continuous distribution. This implies that the hop plant produces a series of PA with particular degrees of polymerisation rather than one main PA with more or less sub units.

### Sedimentation equilibrium (SE) AUCF analysis of proanthocyanidin hop extracts

**Figure 3:**
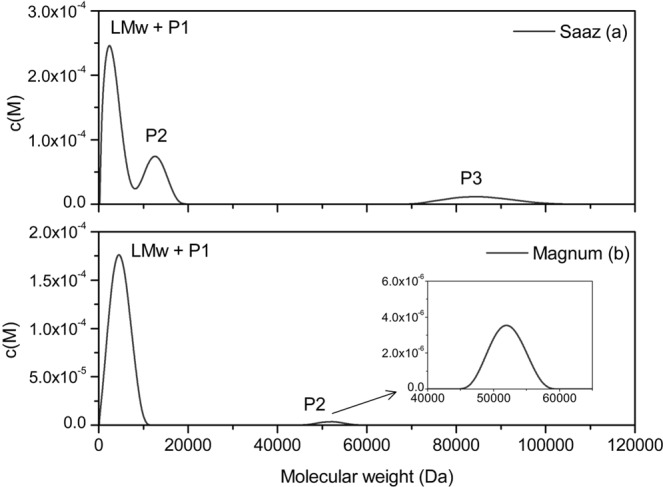
SEDFIT-MSTAR analysis of Sedimentation Equilibrium data, showing the c(M) molecular weight distribution of (**a**) Saaz and (**b**) Magnum hop polyphenolic extracts (40-fold diluted, in methanol). Centrifuged to equilibrium at ascending speeds between 20,000–40,000 rpm at 20 °C.

Sedimentation equilibrium studies can be used to measure the average molecular weight of material in a sample. Although not as resolving as sedimentation velocity, it can provide an absolute estimate of the molecular weights of the major components present. Since the concentration distribution at equilibrium does not change with time, shape and frictional effects do not influence the pattern, which is solely a function of molecular weight. The molecular weight distribution of methanolic SPE fractions derived from Saaz or Magnum hop varieties was studied through sedimentation equilibrium experiments, starting with a low speed of 20000 rpm before moving to higher speeds. Once equilibrium was reached at each speed the rotational speed was increased by increments of 5000 rpm, up to a maximum of 40000 rpm for the final scans. The sedimentation equilibrium studies were consequently of longer duration, taking 4 to 5 days for each run, as compared to the sedimentation velocity experiments which were typically conducted in less than a day. This longer time period is necessary for the sedimentation and diffusive forces to reach equilibrium. Results for Magnum hop variety showed the presence of two distinct peaks, one with a molecular weight average of 5 kDa (97% of total area), and the second peak with a molecular weight of 52 kDa which only accounted for <3% of total area (Fig. 3b). In contrast, the Saaz plot in Fig. 3a has 3 peaks. The first peak has an average molecular weight of 3 kDa (62% Area), the second is at about 12 kDa (26% Area), and lastly there is a higher molecular weight peak at about 85 kDa (12% Area). Clearly the Saaz hop extract contained a greater proportion of higher molecular weight species, which is in agreement with the sedimentation velocity data (Fig. 2).

## Overall Discussion

Polyphenol-rich hop extracts are of interest for their potential to clarify green beer efficiently^4^, but also for their various beneficial impacts on human health.

Degree of polymerisation is an important parameter to take into account in order to extract the highest molecular weight fraction from botanical sources. As the degree of polymerisation of proanthocyanidin polymers increases, they become more hydrophilic^6^. The most concentrated proanthocyanidin fraction was extracted from hops using 70% acetone (aq) which, not surprisingly, correlates with the solvent composition typically recommended for the extraction of proanthocyanidins^23^. This fraction also gave the most efficient results in terms of beer fining activity.

Analytical ultracentrifugation is a well-known method for the determination of the molecular weight of molecules such as proteins and polysaccharides. The sedimentation velocity technique has the ability to profile solutions, to determine the complexity of a mixture, and to give a first indication of approximate molecular weight through the sedimentation coefficients obtained^14^. The results in Table 2 indicate that all of the hop extracts analysed had a heterogeneous molecular weight distribution with peaks at discrete size ranges rather than forming a continuous size distribution. It should be noted that unlike other systems, such as aminocelluloses^24^, no simple single mathematical correlation was found between the sedimentation peaks using the power law relation (s α M^b^) which would apply to species of different molecular weight of similar shape or conformation.

Sedimentation equilibrium was used in this study to resolve and determine actual molecular weights for each band of proanthocyanidins in the hop extracts. The c(M) distributions (Fig. 3) indicated the presence of three different molecular weight groups in Saaz and two in Magnum hops. It is noticeable that in each case the fitting procedure has identified one less peak in the c(M) fit (Fig. 3), relative to the c(s) fit from sedimentation velocity experiments (Fig. 2). It is considered that the lowest mass peak on the c(M) fits correlates with the two lowest sedimentation coefficient peaks on the c(s) fits. This is in part because the resolving power of sedimentation equilibrium experiments is less than that for sedimentation velocity experiments, due to the shorter cells/path lengths used to ensure that equilibrium can be achieved within a reasonable time frame.

The highest mass peak for Saaz was found during sedimentation velocity experiments at 4.4S, which from the data in Fig. 3a corresponded to a mass of around 90 kDa. In contrast, we have the presence of components at about 12 kDa, for a sedimentation peak at 2.6S, suggesting the larger species are more compact structures.

The development of this analytical technique using AUCF has confirmed the presence of very high molecular weight polymers of proanthocyanidins in hops. Despite their low concentrations (approx. 20 mg/mL dry matter in the C18 purified methanolic fractions), AUCF was able to detect this group of polymers. Li *et al*.^25^ have clearly shown the polymer distribution of hop proanthocyanidins with accurate molecular masses, up to hexamers using tandem mass spectrometry. However, due to the noted difficulties in analysing high molecular weight proanthocyanidins, the present results are the first indication that much larger polymers are present in hops. Significantly, AUCF is a non-destructive method which measures the transport properties of molecules through a known distance under centrifugal force.

The possibility remained that high mass species monitored using the AUCF technique might be artefacts of smaller proanthocyanidins bound to other molecules, such as protein or lignin materials from the hop. To investigate this hypothesis we analysed the protein content of the crude 70% acetone (aq) extract using a CB-X assay (G-Biosciences). This indicated a low protein content of less than 1% of dry matter (0.5–0.6 g/L protein out of a total dry matter of 60 g/L). This would not be enough to contribute the high mass peaks observed which corresponded to around 35% of the material present in the Saaz hop (Table 2). Furthermore, we subjected a lignin standard material (Sigma-Aldrich lignin, alkali, low sulfonate; CAS 8068-05-1) to the SPE clean-up procedure used to prepare samples for AUCF and observed almost 0% recovery of lignin (A_280 nm_) into the eluted fraction for analysis. We thus considered that lignin was unlikely to be a significant component of the analysed polyphenolic fraction. Lastly in this regard, if binding between proanthocyanidins and a heterogeneous polymer such as lignin was envisaged, it would seem unlikely that the AUCF sedimentation velocity profiles of 3 different samples of Saaz hop would be so similar to one another as is the case for the results presented in Table 2. We conclude on the basis of the above and the selectivity of the extraction and clean-up procedures that the high molecular weight components are indeed proanthocyanidins. In this regard it can be noted that Tagashira *et al*. reported the presence of ‘high molecular weight polyphenols’ in hop bract extracts (species not specified), estimated to be 36–40 kDa in size^26^.

A significant disadvantage of using chromatographic techniques in proanthocyanidin analysis is the lack of polymer reference standards in a high purity form. Progress has been made in the preparation of synthetic proanthocyanidins^27^, with Ohmori *et al*.^28^ reporting the assembly of catechin monomers to yield a 24-mer. However, this is currently the upward size-limit achieved through synthesis.

AUCF has not previously been applied to the characterisation of proanthocyanidin polymers. This study thus represents a significant step forwards in developing methods for the analysis and characterisation of proanthocyanidins and may ultimately lead to an improved understanding of proanthocyanidin structures and molecular size distributions across the plant kingdom.

## Conclusions

This study has reported for the first time the application of AUCF to the characterisation of polyphenolic extracts of hops, and shown the presence of high molecular weight compounds with an upwards size limit in the region of 100 kDa for Saaz (or 56 kDa for Magnum). The polyphenolic hop extracts prepared in this study comprise a diverse group of compounds. Crude extracts in 70% acetone (aq) contained approximately 60 mg/mL of dry matter. Following SPE clean-up this was reduced to around 20 mg/mL of dry matter in the fractions analysed by AUCF. Importantly, this fraction retained the majority of proanthocyanidins, as indicated by the acid butanol assay. However, it is clear from the AUCF analysis that material of a range of molecular weights was present, which would have comprised not only proanthocyanidins, but also monomeric phenolic acids, polyphenols and potentially tannic acids. Although AUCF is normally applied to homogeneous systems, we have demonstrated that it is possible to adapt this technique to the analysis of such complex mixtures. Sedimentation velocity experiments provided evidence of different sedimentation coefficient profiles when comparing extracts of different hop varieties. These were reproducible when different sources of the same hop variety (Saaz) were analysed.

AUCF is a suitable technique for the non-destructive analysis of high molecular weight proanthocyanidins. As these molecules are also of considerable interest for their beneficial health aspects^29^, this methodology should have cross-disciplinary applications in the characterisation of proanthocyanidins from a range of different sources.

## Data Availability

The datasets generated during and/or analysed during the current study are available from the corresponding author on reasonable request.
